# Do viscosity and wettability of fluoride varnishes affect their fluoride release?

**DOI:** 10.4317/jced.56985

**Published:** 2021-03-01

**Authors:** Jackeline Asian, Edgar Quenta, Jorge L. Castillo

**Affiliations:** 1 Professor, Universidad Peruana Cayetano Heredia, School of Stomatology, Department of Dentistry for Children and Adolescents,Lima, Peru

## Abstract

**Background:**

There are several brands of fluoride varnishes in the market, but the dynamics of fluoride release from each one might be different. The purpose of this study was to evaluate in vitro the release of fluorides by fluoride varnishes and to determine the correlation with viscosity and wettability.

**Material and Methods:**

Forty four enamel blocks 5x5 mm were randomly divided into 4 groups (n=11) ((Duraphat®, Clinpro™ White Varnish, Flúor Protector® and control). We applied 30 milligrams of fluoride varnish to each specimen. The specimens were immersed in a Calcium Phosphate solution at a pH= 6.0. We evaluated the release of fluoride, by using a selective fluoride electrode, during 6 weeks. Viscosity was measured using an Oswald Viscosimeter and the wettability was determined by measuring the contact angle between the varnish and the enamel slab. The statistical analysis was performed using Analysis of variance.

**Results:**

Duraphat showed the highest fluoride release from the second weekend beyond (*p*<0.001) and Clinpro the greatest rate of release. Duraphat release was the steadiest throughout the experiment. Duraphat showed the highest viscosity and the lowest wettability (*p*<0.001) and Fluor Protector showed the highest wettability (*p*<0.001). There was a positive correlation between the release of fluoride and the viscosity and a negative correlation between fluoride release and wettability (r>0.7).

**Conclusions:**

Viscosity and wettability influence the release of fluoride from fluoride varnishes.

** Key words:**Fluorides topical, viscosity, wettability.

## Introduction

Fluoride varnishes are professionally applied fluoride vehicles. According to the latest systematic reviews, the caries reduction by using fluoride varnish is 43% in permanent teeth and 37% in primary teeth, the greatest of all professionally applied fluoride vehicles (1). Fluoride varnishes also have a high performance in preventing and treating white spot lesions during orthodontic treatment (2) and treating incipient caries lesions (3). The explanation for this high performance of fluoride varnishes may be that, compared to other fluoride vehicles, they may adhere to the enamel and other oral surfaces maintaining appropriate levels of fluoride for a prolonged period of time and acting as a reservoir of fluorides to be used when needed (4,5).

There are several commercially available brand names of fluoride varnishes with different active ingredients, composition, technologies and properties (6,7). The American Dental Association recommends the use of Fluoride Varnish with 2.26% Fluoride Ion or 5% Sodium Fluoride (ADA) (8).

The release of fluoride from fluoride varnishes may vary according to the type of varnish (9,10). Our hypothesis is that these variations may be due to their composition, type of resin and other properties, that have not been evaluated yet. Viscosity is the measure of the resistance to flow that a fluid offers when it is subjected to shear stress (11). It seems reasonable that a varnish that is more viscous will stay longer over a tooth surface, therefore it will release fluoride for a longer period of time. Wettability is the ease or otherwise of a liquid to spread over a surface (12). The most common method of observing wetting is measuring the contact angle. The contact angle is the internal angle in a droplet of liquid in contact with a solid (12). We may also speculate that the wider the spread of the varnish, the higher chance to cover all the areas of the teeth surfaces.

There are no studies that have evaluated the correlation between fluoride release from fluoride varnishes and their viscosity and wettability. The aim of this study is to evaluate the fluoride release from 3 commercially available fluoride varnishes and correlate it with its viscosity and wettability.

## Material and Methods

-Sample calculation.

For sample size calculation, we used the formula of the difference between the means of two samples, from the pilot study. The pilot study was done with 10% of the sample size of a previous study5 obtaining a sample of 9 specimens per group. We used the sample size calculation adjusted to specimens lost during the experiment, ending up with 11 specimens per group.

-Study design. This is an experimental in-vitro study. We obtained enamel slabs of 5x5 mm from healthy premolars extracted for orthodontic reasons. We previously cleaned and disinfected the teeth. The specimens were selected and randomly assigned to one of 4 groups: Duraphat (Colgate-Palmolive, New York, NY, USA), Clinpro White Varnish (3M ESPE, MN, USA), Fluor Protector (Ivoclar Vivadent, Amherst, Nueva York, USA) and Varnal as a control group (Biodinámica, Paraná, Brasil). Eleven samples were assigned to each group.

-Specimen preparation. The teeth were cut with a Double Sided Diamond Disc 916DF-220 fine (Henry Schein International, USA) in order to obtain an enamel slab of 5 x 5 mm. The slabs were measured with a digital caliper (Mitutoyo, Tokio, Japan). We placed one end of a piece of dental floss over the dentin surface of each slab and covered it with a photo-cured resin Z350™ A3 Body (3M ESPE, MN, USA) in order to handle the specimen more easily.

-Varnish application procedure. Thirty milligrams of fluoride varnish were placed on each specimen. That means that for Duraphat and Clinpro 37.5 umol of fluoride and for Fluor Protector 1.58 umol of fluoride were applied.

After the application, each specimen was immersed in 20 ml of a buffer Calcium Phosphate solution (pH 6.0) at room temperature 5. The other end of the dental floss was used to secure the sample inside the tube.

Each day during the first week and then each week for the remaining 6 weeks, a new test tube was prepared with 20 milliliters of the calcium phosphate solution. We transferred the samples to the new tubes and the old ones were analyzed for their fluoride content.

-Fluoride release. The fluoride analysis was performed in the Fluoride Analysis lab of the University XXXXXX. We used the ion analyzer (Versa Star A329, Orion, Thermo Scientific) and a fluoride selective electrode (Plus Model 9606 VPN, Orion, Thermo Scientific). We calibrated the electrode with TISAB (total ionic strength adjusting buffer) and fluoride standards. A new calibration curve was done with fresh standards before every day the solution was measured.

Daily during the first week and then once weekly for 6 weeks, we measured the concentration of fluoride in the solution to determine the amount of fluoride released by the sample. Millivoltage was read and converted into parts per million using the calibration curves. One of the investigators (JA) did all the measurement and did not know which sample belonged to which group. The electrode was constantly calibrated to avoid any bias due to the malfunctioning of the instrument.

-Rate of fluoride release. The rate of fluoride release was calculated for the mean slope of each of the products 5,9. We calculated the rate of fluoride release between day 1 to 7.

-Viscosity analysis. The viscosity analysis was done in the Physics Analysis Lab at the University YYYYYYY, using an Ostwald Viscometer (Sigma-Aldrich Corporation, St. Louis, MO, USA) and measured at room temperature. Viscosity was determined using the formula: ϒ = (µglicerin x tvarnish / Pglicerin x tglicerin) where: ϒ = varnish viscosity in centistokes, µglicerin = glycerin viscosity, tvarnish= time for the varnish to flow through the tube, tglicerin= time for the glycerin to flow through the tube, Pglycerin= glycerin density. The viscosity is expressed in Centistokes units (cSt).

-Wettability analysis. Wettability analysis was performed by determining the angle of contact that is formed when the liquid contact a solid. First, we measured the width of an enamel block in different zones. We placed the enamel block in glass slides and a drop of varnish was applied using a syringe. After the varnish dried, we measured again the width and the diameter of the enamel block, and the tangent of the contact angle. The tangent of the angle was obtained measuring the diameter (d), the angle of the drop (d) and the height of the drop (h) using the formula: Angle = arctg [h/(d/2)] 

-Statistical analysis. We used ANOVA, Tukey and t-Student for normal data and Kruskal-Wallis, Mann-Whitney U and Wilcoxon for non-normal data, to compare the average fluoride release, cumulative fluoride release, viscosity and wettability of the varnishes. We used the Pearson correlation to evaluate the correlation between the release of fluorides and the viscosity and wettability. We also used the determination coefficient to evaluate the strength of the correlation.

## Results

-Fluoride release. The average of concentration of fluoride release (ppm) during the first 7 days and then weekly during 6 weeks are presented in Table 1. All the varnishes showed a reduction in the release of fluoride during that period of time.

**Table 1:**
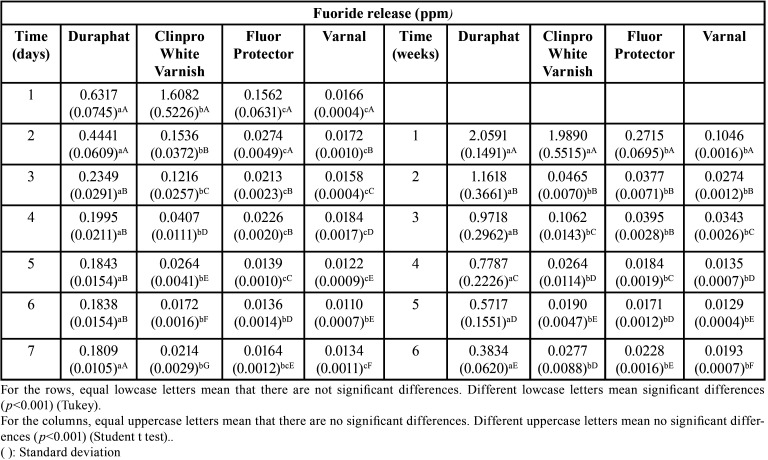
Fluoride release (ppm) from fluoride varnishes during 7 days and 6 weeks.

From day 1 to day 7

Clinpro showed the highest release of fluoride during the first day (*p*<0.001). From day 2 to day 7, Duraphat showed the highest release of fluoride followed by Clinpro (*p*<0.001). Fluor Protector and Varnal did not show any differences in the release during the first 7 days (*p*>0.05).

From week 1 to week 6

Duraphat showed the highest release of fluoride during all the experiment (*p*<0.001), except week 1, where there no differences between Duraphat and Clinpro (*p*>0.05). From week 2 and beyond, Clinpro, Fluor Protector and Varnal did not show any difference in fluoride release (*p*>0.05).

-Cumulative release of fluorides. All the varnishes showed a significant increase of the cumulative release during the whole period of this experiment (*p*<0.001).

From day 1 to day 7

Clinpro showed the highest cumulative release of fluoride during the first 4 days (*p*<0.001). From day 5 to day 7, Duraphat and Clinpro did not show any significant differences (*p*>0.05).

From week 1 to week 7

From week 2, Duraphat showed the highest cumulative release (*p*<0.001), followed by Clinpro, Fluor Protector and Varnal. There were no significant differences between Fluor Protector and Varnal throughout the experiment (*p*>0.05).

-Rate of fluoride release.

**Figure 1:**
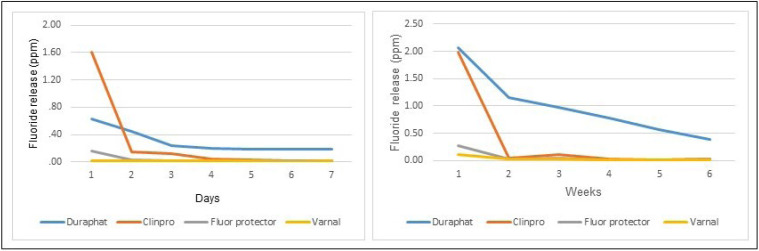
Release of fluoride varnishes during 7 days and 6 weeks.

The rate of release, which is the average slope, was calculated between day 1 and 7 (Table 2). The highest rate of release was observed with Clinpro, meanwhile Varnal had a very slow rate of fluoride release followed by Fluor Protector and Duraphat (Fig. 1).

**Table 2:**
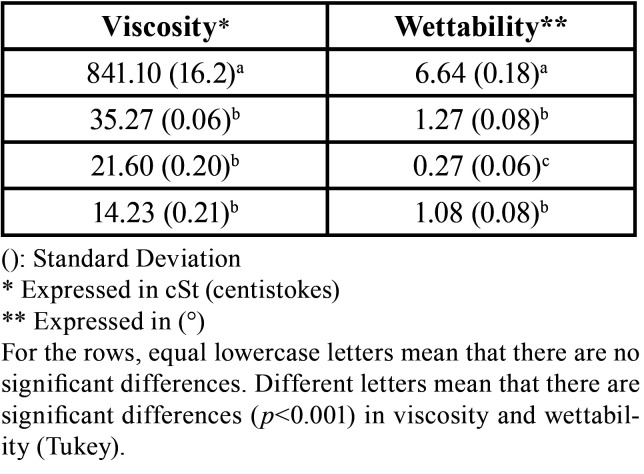
Rate (slope) of fluoride release during the first 7 days and viscosity and wettability of the varnishes.

-Viscosity and wettability. The highest viscosity (841.1 cSt) and the lowest wettability (greatest angle of contact: 6.64°) was found with Duraphat. The lowest viscosity was found with Varnal (14.23 cSt) and the highest wettability (lowest contact angle: 0.27°) in Fluor Protector (Table 2).

There was a high positive correlation (r>0.7) between fluoride release and viscosity and there was a high negative correlation (r>0.8) between fluoride release and wettability of fluoride varnishes (Table 3).

**Table 3:**
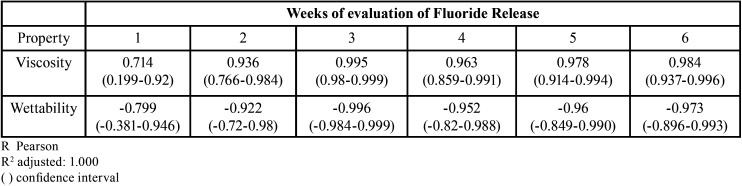
Correlation between fluoride release from fluoride varnishes with viscosity and wettability during 6 weeks.

## Discussion

Previous studies found differences in fluoride release when comparing different fluoride varnishes (5,9). The question is whether this difference can be due to differences in the physical properties of the products. And that is the reason why we planned this investigation.

We tested the products that have more presence in the market. In the last systematic review on fluoride varnishes, more than 60% of the selected articles used Duraphat from Colgate as the varnish tested (1). The comparison was done with other two popular products: Clinpro from 3M and Fluor Protector from Ivoclair Vivadent.

The mode of release was different among the varnishes during the experiment. During the first 7 days, the highest release rate was observed in Clinpro and Duraphat compared to Fluor Protector and Varnal. From week 2 to the end of the experiment, Duraphat showed the greatest release of fluoride. This is an interesting finding; both Clinpro and Duraphat have the same active principle (5% Sodium Fluoride), but they behave different. Duraphat had the advantage of higher fluoride cumulative release throughout most of the experiment.

The speed and fluoride release behavior differ significantly according to the type of varnish and decreased over time. This is in accordande with Shen and Autio Gold (13) who reported that the release of fluorides in the first week, and even more in the first 7 hours, seems to be faster for every fluoride varnish studied. Also Castillo (5) found that Duraphat had a higher cumulative release and a more prolonged fluoride release compared to Duraflor.

These differences observed between the varnishes may be due, as has already been described in the literature, to the differences in the type of base present in its composition and physical properties, or other aspects of the fluoride varnishes formulation which had not been evaluated so far. Duraphat and Clinpro are varnishes that have a natural resin base and Fluoride Protector a polyurethane base, according to the manufacturers. Maas JR *et al.* (14) reported that fluoride ion diffusion process is slower in fluoride varnishes with a natural resin base. This may explain the greater release of fluorides from varnishes with this base. Downey *et al.* (15) found that the formulation of other ingredients in fluoride varnishes can affect the fluoride concentration in saliva. Every fluoride varnish brand may use different components that may affect their performance.

Another important aspect is the presentation of the products. While Duraphat presentation is in 10 ml tubes, Clinpro and Fluor Protector are presented in unit doses. In both presentations, the components of the varnish are separated with time and show a non-uniform distribution of the fluoride content, so the manufacturers recommend rubbing (Duraphat), mixing (Clinpro) or shaking (Fluor Protector) before applying it to homogenize the fluoride content, however this may change the fluoride content for each type of varnish.

We hypothesized that the physical properties of the products may be the reason for the difference in the fluoride release observed in each product. Two physical properties of the varnishes were studied and correlated with the fluoride release: viscosity and wettability.

In this study, as in a previous one (5), the amount of varnish applied (30 mg) was the same for all the specimens of the different groups, so it was possible to make comparisons between them. However, the weighing of Fluor Protector, a varnish that volatilizes quickly due to the solvent, was difficult. Maas JR *et al.* (14) reported in their study that the handling of most of the fluoride varnishes studied was difficult due to the different physical characteristics that could be influenced by the viscosity of the varnish, base type, among other characteristics as was also mentioned by Bolis C *et al.* (7) making difficult to measure the quantity dispensed with great precision. Also, 30 milligrams of Fluor Protector, contains a smaller amount of fluoride compared to Duraphat and Clinpro.

Viscosity is an important property of pharmaceutical products and food. Studies have found that viscosity has an influence on the way some drugs release their active principle (16,17), increasing their effectivity. For instance, Studies have found a reciprocal correlation between the viscosity of ointments and the quantity of the released drugs (18). Other studies have found that the aroma release and intensity olfactory perception were stronger in low-viscosity yogurts than in high-viscosity yogurts (19).

When studying the viscosity of fluoride varnishes, it was found that it differs between varnishes as was also reported by Bolis C *et al.* (7) who found that Fluor Protector had the lowest viscosity. However, the authors did not use measuring instruments to measure the viscosity of the varnishes. This result was also found in our study using the viscometer as a measuring instrument. Duraphat was the varnish that showed the highest viscosity and the highest release of fluorides. By increasing the viscosity, the ions cannot move freely due to their attractive forces (20), therefore there would be release of fluorides for a longer time. Other studies have found a relation with viscosity and fluoride release from Fluoride varnishes. Pichaiaukrit W *et al.* (21) found , in an invitro experiment, that by adding chitosan, which makes varnish more viscous, the release of fluorides is increased.

Wettability is also an important property that has been studied in drugs. The larger the area of contact of a specific drug, the larger the area of effect. Some studies have found a correlation between the wettabilty and the drug efficacy (22,23). Differences between varnishes were found when studying wettability by contact angle. The highest wettability was observed in Fluor Protector followed by Clinpro and Duraphat, which coincides with the data from lower to higher fluoride release of the fluoride varnishes respectively.

Fluor Protector presented the highest wettability, the lowest viscosity but also the lowest fluoride release. Initially, a fluoride varnish may cover an extensive area of a tooth (high wettability), but it may wear way very fast, which may not help in the long-term release of fluoride. We have to remember the wettability depends on the liquid but also on the surface were the liquid is deposited. There are many events over the tooth surface (saliva, plaque, mastication, etc), that may affect or minimize the wettability of the varnish.

According to our findings, the most important property of fluoride varnishes is viscosity. As there is a negative correlation between wettability and viscosity, a greater wettability can be negative in the behavior of the fluoride varnishes. Nevertheless, a very viscous varnish, but capable of covering the greatest amount of surface (high wettability), could be ideally the best option and allow fluoride to be released for a longer time, covering the largest amount of teeth surfaces. Other studies have found the same relation between viscosity and wettability. Park *et al.* (24) found a negative correlation between viscosity and wettability when studying human saliva. A study using varnishes with different viscosities and wettabilities may be helpful to find the ideal varnish in terms of physical properties that can assure the most efficient fluoride release throughout time. In addition, further studies are needed to evaluate the correlation between the release of fluorides from fluoride varnishes and other properties of the product such as pH, surface tension, temperature variations to complement this research. It is important to understand the fluoride varnishes physical properties that may influence the release of fluorides in order to find the most effective fluoride varnish for the prevention and control of the progression of dental caries.

## Conclusions

1. Viscosity and wettability influence the fluoride release from fluoride varnishes. According to our findings, higher viscosity and lower wettability of fluoride varnishes is related to a greater release of fluorides.

2. Duraphat presented the highest release of fluorides and the highest viscosity and lowest wettability.
